# tRNA modifications as regulators of bacterial virulence and stress responses

**DOI:** 10.1371/journal.ppat.1013600

**Published:** 2025-10-23

**Authors:** Chloé Teixeira, François Vandenesch, Karen Moreau

**Affiliations:** 1 CIRI, Centre International de Recherche en Infectiologie, Université de Lyon, Inserm U1111, Université Claude Bernard Lyon 1, CNRS UMR5308, ENS de Lyon, Lyon, France; 2 Centre National de Référence des Staphylocoques, Institut des agents infectieux, Hospices Civils de Lyon, Lyon, France; Monash University, UNITED STATES OF AMERICA

## Abstract

Bacteria are recognized for their ability to adapt their lifestyle to the environment. Specifically, when considering pathogenic bacteria, their capacity to respond to stress and switch to a virulent state through gene regulation is crucial. One of the mechanisms that enables regulation of gene expression at the translational level is RNA modification. These chemical changes produced by specific enzymes are present on all types of RNAs and can modulate translational efficiency by influencing the structure of RNA molecules, the codon usage bias, the interaction with other molecules, or the efficiency of ribosome action. Transfer RNA (tRNA) is the most modified RNA in the cell, with modifications in the core body of the tRNA primarily affecting the stability and flexibility of the structure while modifications in the anticodon stem-loop (ASL) are more involved in decoding, as well as the efficiency and fidelity of translation. Given the impacts of these modifications on the translation process and the critical role of modulating translation fidelity during bacterial stress responses and host interactions, tRNA modifications play an important role in regulating the expression of virulence factors in bacterial pathogens, resulting in changes in various phenotypes. This review aims to establish a comprehensive landscape of tRNA modifications and their direct impact on the translation process, emphasizing their significant role in bacterial virulence and stress responses.

## Introduction

Pathogenic bacteria are responsible for various infections and quickly adapt to different environments thanks to the numerous virulence factors they possess [1,2]. These virulence factors are finely regulated to be produced at the right time and place during infection thanks to various regulatory systems that impact gene expression at different levels. At the transcriptional level, transcription factors or epigenetic modifications that target DNA are the main actors; at the post-transcriptional level, an important mechanism is the alteration of messenger RNA (mRNA) translation by regulatory RNAs that impact both the structure and stability of mRNA [3]. Finally, at the translational level regulatory systems can modulate the translation process thanks to regulatory RNAs, riboswitches, ribosomal proteins, and RNA modifications [4].

RNA modifications, first discovered in eukaryotes and subsequently in prokaryotes, are present on all types of RNA and can be added or removed by specific modifying enzymes [5]. Since the modifications are present on transfer RNAs (tRNAs) and ribosomal RNAs (rRNAs), which play a central role in ensuring the fidelity and efficiency of protein synthesis [6], they are involved in modulating translation efficiency and can alter or enhance protein production [7]. tRNA modifications represent a system for pathogenic bacteria to regulate gene expression, particularly in shaping the virulence of bacterial pathogens by impacting the production of virulence factors and stress response proteins. Herein, we illustrate the diversity of tRNA modifications in bacteria, their direct impact on the translation process, and how they regulate the virulence program and stress responses during bacterial infection.

## Variety and complexity of tRNA modifications in bacteria

The identification of the first DNA modification in eucaryotes was made in 1950 with the finding of a non-canonical base (5-methylcytosine) [8]. This was followed closely by the discovery in 1956 of the first RNA modification in yeast with the transformation of uridine in pseudouridine [9], and there has since been a steady increase in the number of new types of RNA modification [5]. To date, a total of 433 different modified RNA residues have been identified in the organisms of the three domains of life, including 71 modifications specific to eukaryotes, 17 to archaea, and 68 to bacteria; the remaining 277 are common to all three domains, as listed in the MODOMICS database. tRNA modifications represent slightly more than half (53.6%) of these RNA modifications [7].

There is an increased interest in studying tRNA modifications owing to their role in bacterial physiology. Thus, recent reviews have primarily focused either on specific tRNA modifications [10,11] or on the functional consequences of these modifications on the translation process [12]. Other recent contributions have highlighted the technical challenges associated with studying tRNA modifications [13], or have provided broader overviews of RNA modifications across different RNA species [14,15]. Given the rapidly evolving knowledge in this field, we present herein an update on the tRNA modifications identified in bacteria, with a focus on pathogenic bacteria, and illustrate their notable impacts with various examples.

### Which modifications are present on bacterial tRNA?

A diverse range of modifications can be made to tRNAs and the first glossary cataloguing these modifications was published in 1994 by Limbach *and colleagues* [16]. In the domain of bacteria, these modifications, which can be permanent or transitory, vary from elementary chemical alterations to more intricate modifications involving the incorporation of complex groups (Table 1).

**Table 1:** Summary of bacterial tRNA modifications and their associated enzymes.

Enzymes	Bacterial species	Modification^*^	Definition
Thil	*E. coli*, *V. cholerae*	s^4^U8	4-thiouridine
TruD	*E. coli*	ψ13	pseudouridine
DusC	*E. coli*	D16	dihydrouridine
DusB1/DusB2	*E. coli*	D17	dihydrouridine
TrmH	*E. coli*	Gm18	2′-O-methylguanosine
DusA/DusB1/DusB2	*E. coli*	D20	dihydrouridine
DusA/DusB1/DusB2	*E. coli*	D20a	dihydrouridine
TapT	*V. cholerae*	acp^3^U20b	3-(3-amino-3-carboxypropyl)-uridine
AcpA	*V. cholerae*	acacp^3^U20b	acetylated 3-(3-amino-3-carboxypropyl)-uridine
TrmK	*B. subtilis*	m^1^A22	1-methyladenosine
TrmJ	*P. aeruginosa*	Am32	2′-O-methyladenosine
TrmJ	*P. aeruginosa*	Cm32	2′-O-methylcytidine
TrmJ	*P. aeruginosa*	Um32	2′-O-methyluridine
TtcA + IscS	*E. coli*, *P. aeruginosa*	s^2^C32	2-thiocytidine
RluA	*E. coli*	ψ32	pseudouridine
TrmL	*E. coli*	Cm34	2′-O-methylcytidine
TilS	*V. cholerae*	ava^2^C34	2-aminovaleramididine
TmcA	*E. coli*	ac^4^C34	N4-acetylcytidine
TilS	*E. coli*, *B. subtilis*, *V. cholerae*	k^2^C34	2-lysidine
TrmL	*E. coli*	Um34	2′-O-methyluridine
TusA/MnmA	*E. coli*, *M. tuberculosis*	s^2^U34	2-thiouridine
TadA	*E. coli*, *B. subtilis*, *S. aureus*	I34	inosine
QueDECFAG(H [32]) + Tgt	*E. coli*, *B. subtilis,* *V. cholerae*, *S. flexneri*	Q34	queuosine
GluQRS	*E. coli*	GluQ34	glutamyl-queuosine
MnmE + MnmG^**^	*E. coli*, *P. aeruginosa*	nm^5^U34	5-aminomethyluridine
MnmE + MnmG	*E. coli*	mnm^5^U34	5-methylaminomethyluridine
MnmE + MnmG	*E. coli*, *P. aeruginosa, * *S. pyogenes*	cmnm^5^U34	5-carboxymethylaminomethyluridine
MnmA + MnmE + MnmG (+ MnmC/MnmL [33])	*E. coli*, *B. subtilis*, *P. aeruginosa*	nm^5^s^2^U34	5-aminomethyl-2-thiouridine
MnmA + MnmE + MnmG + MnmC/MnmM	*E. coli*, *B. subtilis*, *S. enterica*	mnm^5^s^2^U34	5-methylaminomethyl-2-thiouridine
MnmA + MnmE + MnmG	*E. coli*, *B. subtilis*, *P. aeruginosa*	cmnm^5^s^2^U34	5-carboxymethylaminomethyl-2-thiouridine
MnmE + MnmG + TrmL	*E. coli*	cmnm^5^Um34	5-carboxymethylaminomethyl-2′-O-methyluridine
CmoM + TrmL	*E. coli*	mcmo^5^Um34	2′-O-methyluridine 5-oxyacetic acid methyl ester
MnmA + MnmE + TrmL	*E. coli*	mnm^5^s^2^Um34	5-methylaminomethyl-2-thio-′-O-methyluridine
TrmR	*B. subtilis*	mo^5^U34	5-methoxyuridine
CmoA + CmoB	*E. coli*, *M. bovis*	cmo^5^U34	uridine 5-oxyacetic acid
CmoM	*E. coli*	mcmo^5^U34	uridine 5-oxyacetic acid methyl ester
ThrP/ThrO	*E. coli*	ho^5^U34	5-hydroxyuridine
RluF	*E. coli*	ψ35	pseudouridine
TrmD	*E. coli*, *B. subtilis*, *S. enterica*	m^1^G37	1-methylguanosine
RlmN	*E. coli*	m^2^A37	2-methyladenosine
TrmM	*E. coli*	m^6^A37	6-methyladenosine
TsaBCDE	*E. coli*, *B. subtilis*, *P. aeruginosa*	t^6^A37	N6-threonylcarbamoyladenosine
TcdA	*E. coli*	ct^6^A37	cyclic N6-threonylcarbamoyladenosine
TrmO	*E. coli*	m^6^t^6^A37	N6-methyl-N6-threonylcarbamoyladenosine
MiaA	*E. coli*, *S. flexneri*	i^6^A37	N6-isopentenyladenosine
MiaB	*E. coli*, *S. flexneri*	ms^2^i^6^A37	2-methylthio-N6-isopentenyladenosine
MiaE	*S. typhimurium*	ms^2^io^6^A37	2-methylthio-N6-(cis-hydroxyisopentenyl)-adenosine
MtaB	*B. subtilis*	ms^2^t^6^A37	2-methylthio-N6-threonylcarbamoyl-adenosine
TruA	*E. coli*	ψ38	pseudouridine
TruA	*E. coli*	ψ39	pseudouridine
TruA	*E. coli*	ψ40	pseudouridine
TrmB	*E. coli*, *A. baumannii*	m^7^G46	7-methylguanosine
TapT	*V. cholerae*	acp^3^U46	3-(3-amino-3-carboxypropyl)-uridine
AcpA	*V. cholerae*	acacp^3^U46	acetylated 3-(3-amino-3-carboxypropyl)-uridine
DusB1	*B. subtilis*	D47	dihydrouridine
TapT	*E. coli*, *V. cholerae*	acp^3^U47	3-(3-amino-3-carboxypropyl)-uridine
AcpA	*V. cholerae*	acacp^3^U47	acetylated 3-(3-amino-3-carboxypropyl)-uridine
RsmF	*E. coli*	m^5^C49	5-methylcytidine
TrmA	*E. coli*	m^5^U54	5-methyluridine
TruB	*E. coli*, *S. flexneri*	ψ55	pseudouridine
TrmI	*M. tuberculosis*	m^1^A58	1-methyladenosine
TruC	*E. coli*	ψ65	pseudouridine

The Modomics database is the main source of the data listed in this table [7]. The bacterial species column contains data mainly extracted from Modomics database, as well as information on bacterial models and pathogens discussed throughout this review.

* The number indicates the position of modification on tRNA.

** MnmG was previously named GidA and can be referenced as such in the literature.

Nucleotide methylation is the most common type of modification, as it occurs on all four canonical nucleotides. Regarding the nitrogenous bases, adenine can be methylated at various positions (m^1^A, m^2^A, and m^6^A) as can guanine (m^1^G, m^2^G, and m^7^G), whereas uridine and cytosine are only modified at one position (m^5^U and m^5^C). Methylation also occurs on the ribose part of the four nucleotides, this process is known as a 2′-O-methylation (Am, Gm, Um, and Cm) [10,17,18].

Certain modifications are specific to a single type of nucleotide. For example, isomerization of the base to form pseudouridine (ψ) [19] or the hydrogenation to form dihydrouridine (D) [20] are specific to uridine. Moreover, deamination reaction to form inosine (I) [21] and the addition of a threonylcarbamoyl group (t^6^A) [22] are specific to adenosine while the complex modification into queuosine (Q) is specific to guanosine [23]. Similarly, the acylation reaction is specific to cytosine (ac^4^C) [10]. Other modifications involve the addition of a sulfur atom with the thiolation at position 2 for cytidine (s^2^C) [24] and at position 2 or 4 for uridine (s^2^U and s^4^U) [25].

Among this variety of modifications, some are sequential and require the completion of an initial modification prior to their occurrence. This is the case of the methyl thiolation of adenosine (ms^2^i^6^A) that necessitates the previous addition of an isopentyl group to form i^6^A [26]. Similarly, the acacp^3^U modification discovered in *Vibrio cholerae* (*V. cholerae*) is an acetylated form of the acp^3^U modification [27]. Other examples include the successive reactions that modify uridine to create mnm^5^s^2^U and then cmnm^5^s^2^U [28], or the hydroxylation at position 5 to form ho^5^U, followed by the methylation at the same position to produce mo^5^U, and finally the carboxylation to generate cmo^5^U [29]. Notably, certain modifications do not necessitate an initial modification to arise; however, the existence of specific modifications can facilitate their occurrence. For example, in *Escherichia coli* (*E. coli*), acp^3^U47 is stimulated by m^7^G46 and m^5^U54, as is ms^2^i^6^A37 by m^5^U54, and s^4^U8 by ψ55 and m^5^U54 [30].

Bacteria therefore exhibit a diversity of tRNA modifications, some of which are exclusive to their kingdom. Among the modifications cited above, m^2^A, s^2^C, s^4^U, ms^2^i^6^A, mnm^5^s^2^U, ho^5^U, mo^5^U, and cmo^5^U are specific to bacteria [7,10,18,31].

### What achieves these modifications?

Specific enzymes catalyze the chemical reactions to modify tRNAs at precise sites (Table 1). The first tRNA-modifying enzyme was published in 1962 and 1963 by two groups, Borek in the USA and Svensson in Sweden [5]. This enzyme, known in bacteria as TrmA methylates the uridine at position 54 of almost all tRNAs to form m^5^U54 [34,35].

Improvements in the detection of tRNA modifications and next-generation sequencing [36–38], combined with comparative genomics and structural studies, have facilitated the comprehensive mapping of enzymes with the corresponding tRNA modifications in model bacteria such as *E. coli* and *Bacillus subtilis* (*B. subtilis*) [39]. Recently, the predicted landscape of tRNA modifications has been explored and partially validated in other bacteria, such as *Pseudomonas aeruginosa* (*P. aeruginosa*) [40], two Bartonella species [41], and *Streptomyces albidoflavus* [42]. Additionally, tRNA modifications have been identified in *Staphylococcus aureus* (*S. aureus*) [43].

Most enzymes involved in RNA modifications target a single type of RNAs, typically tRNAs or rRNAs, and less commonly other non-coding RNAs and mRNAs. However, there are a few notable exceptions where certain enzymes perform the same type of modification on multiple types of RNA. For example, RlmN in *E. coli* catalyzes m^2^A37 in tRNA and m^2^A2503 in 23S rRNA [44]; and RluA catalyzes the formation of pseudouridine at position 32 in tRNA and 746 in rRNA [39]. Another dual-specificity enzyme is the TadA enzyme that deaminates adenosine to inosine at position 34 of Arg-tRNA and also catalyzes the formation of inosine in bacterial mRNA [21,45]. Interestingly, certain enzymes act specifically on tRNAs but are responsible for two types of modifications. This is the case of TilS in *V. cholerae*, which catalyzes both the k^2^C34 and the ava^2^C34 modifications in tRNA-Ile [46,47].

These enzymes are rather conserved among bacteria and most of the modifications identified so far are shared between species. Nevertheless, a comparison of the tRNA modification landscape of *E. coli* and *B. subtilis* shows that the modifications of tRNAs from Gram-positive bacteria are less diverse than that from Gram-negative bacteria, and some modifications appear to be specific to one of the two species. For instance, ψ55 and Gm18 modifications are only detected in *E. coli* whereas m^1^A22 and ms^2^t^6^A37 modifications are specific to *B. subtilis* [39].

### Which positions on the tRNA are subject to these modifications?

**Fig 1:**
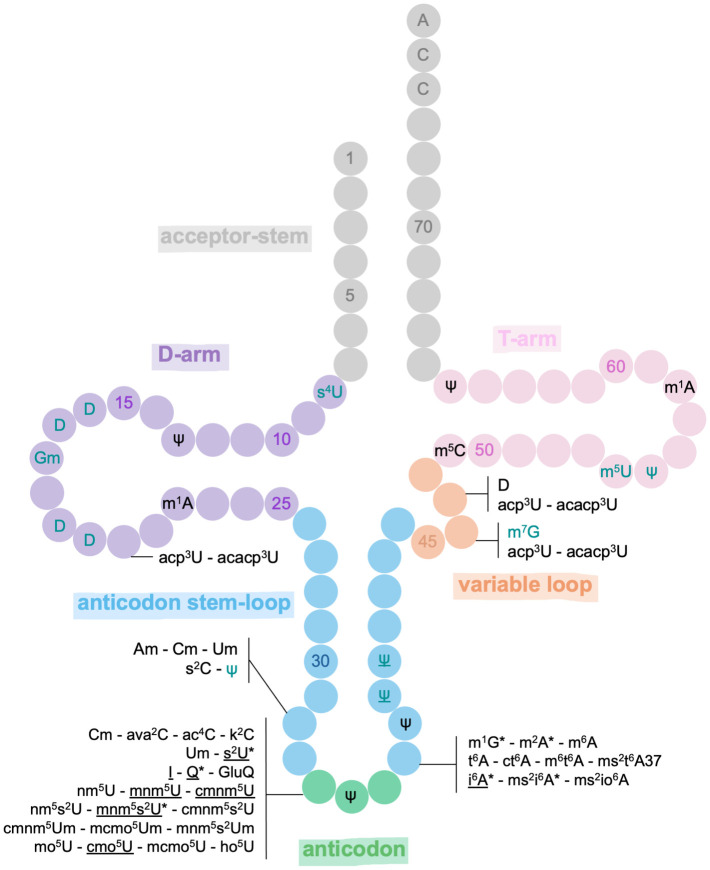
Map of bacterial tRNA modifications and their impact on translation. The secondary structure of tRNA is numbered each five positions and is composed of six parts. The acceptor-stem is colored in gray, the D-arm in purple, the variable loop in orange, the T-arm in pink, the anticodon stem-loop in blue, and the anticodon in green. The effects of the modifications on translation mentioned in this review and previously known are indicated as follows. The modification is in duck blue if it affects tRNA structure; is marked with an asterisk if it affects reading frame maintenance or readthrough; and is underlined if it affects codon decoding.

tRNAs are the most modified RNAs, harboring an average of 14 modifications per molecule, equivalent to one modification every three nucleotides [48]. Among the four major parts of tRNA secondary structure, while the D- and T-arms possess some modifications, the most modified region is the anticodon stem-loop (ASL) within which two residues are hypermodified, the first at position 34 (the wobble base, the third of the anticodon) and the second at position 37 (adjacent to the anticodon; Fig 1). For example, in *E. coli*, 16 distinct modifications have been identified at position 34, while eight have been found at position 37. In *B. subtilis*, 12 different modifications have been detected at position 34, and nine at position 37 [39].

Certain modifications are specific to one region, such as dihydrouridine in the D-arm or inosine and queuosine that are only present at position 34. This indicates that each modification has a specific role and effect on tRNA activity; modifications present in the core body (elbows and arms) of the tRNA are implicated in the stability and flexibility of the structure whereas modifications in the ASL are involved in decoding aspects, as well as efficiency and fidelity of translation [49] (Fig 1). Furthermore, it is important to note that not all tRNAs exhibit all types of modifications, and most modifications are specific to one or a few different tRNAs [7].

## Effects of tRNA modifications on the translation process

Translation, along with replication and transcription, is one of the three fundamental molecular processes essential for cell viability and is composed of four steps. Firstly, translation is initiated with the assembly of the ribosome complex on the produced mRNA at the start codon. Then, the elongation of the new peptide requires the correct decoding of the mRNA codons by tRNA molecules until a stop codon is reached. Termination factors then hydrolyze the ester bond of the peptidyl tRNA to produce the new protein and dissociate the complex for ribosome recycling [6]. A few regulatory systems can modulate the process. For example, some regulatory RNAs, ribosomal proteins, and riboswitches can prevent the formation of the ribosomal complex or induce specific mRNA structural changes to control the initiation step [4]. An additional regulatory mechanism involves tRNA modifications, as they affect tRNAs structure and stability, thereby impacting the fidelity and efficiency of translation, in which tRNAs play a central role [10].

### How tRNA modifications impact tRNA structure?

The structural characteristics of tRNA, including its 2D cloverleaf and 3D L-shaped structure, are crucial for translation, as they allow interactions with mRNA, ribosomes, and aminoacyl-tRNA synthetases. To maintain these structural characteristics, tRNA modifications are required. For example, in mitochondrial tRNA-Lys, the presence of m^1^A9 modification disrupts Watson-Crick base pairing to allow formation of secondary and tertiary structures [50]. In bacteria, 2′O-methylation, thiolation, pseudouridylation [30], and m^5^U modifications [51] stabilize the structure of tRNA by inducing conformational rigidity through an improvement of base stacking interactions [52]. In contrast, dihydrouridine is the only known modification that promotes flexibility of tRNA structure [20]. Additionally, ψ32 and ψ39 are involved in the correct ASL structure of *E. coli* tRNAs [53].

Moreover, it has been demonstrated that certain modifying enzymes have a chaperone function that facilitates the folding of tRNAs such as TruB (ψ) and TrmA (m^5^U) enzymes in *E. coli* that contribute to the correct folding of tRNAs, even in the absence of their catalytic activity [54,55].

### How tRNA modifications affect readthrough and reading frame maintenance?

During translation, recognition of stop codons and maintenance of the reading frame are critical to ensure the accurate protein production. Modifications in the ASL region are important to prevent readthrough. In **E. coli*,* it has been reported that the absence of Q34 or m^2^A37 leads to an increase of readthrough at the UAG stop codon [44,56]. ASL modifications are also crucial for reading frame maintenance. In **E. coli*,* the absence of m^1^G37 in tRNA-Pro results in an incorrect translation of proline, particularly for CCC and CCU codons as this modification prevents the ribosome from stalling on the mRNA, thereby avoiding a + 1 frameshift and premature termination [57,58]. In extraintestinal pathogenic *Escherichia coli* (ExPEC) strains, the amount of i^6^A37 modification catalyzed by the MiaA enzyme modulates −1 and +1 frameshifts; a deletion or over-expression of *miaA* increases frameshifts errors [59]. Another modification at residue 37, ms^2^i^6^A37, prevents frameshifting by stabilizing the interaction between the tRNA anticodon at position 34 and the mRNA codon at position 1 [60]. Similarly to ms^2^i^6^A37, the mnm^5^s^2^U34 modification avoids frameshifting by increasing the affinity of the codon-anticodon interaction [61]. In addition, the formation of queuosine (Q) or the thiolation of uridine (s_2_U) at position 34 participates in reading frame maintenance [60,62].

### How tRNA modifications influence decoding and codon usage bias?

The degeneracy of the genetic code is linked to crucial interactions between the wobble base of the mRNA codon and the anticodon first base at residue 34. These interactions are influenced by tRNA modifications within or near the anticodon, which are essential for ensuring the fidelity of translation by affecting decoding aspects. A notable example in bacteria is the queuosine modification which replaces guanosine at position 34 of tRNA-GUN that decodes NAC and NAU codons (Asp-GAC/GAU, Asn-AAC/AAU, Tyr-UAC/UAU, and His-CAC/CAU). In *E. coli*, G34-tRNAs preferentially decode NAC over NAU codons while Q34-tRNAs exhibit an enhancement of translational efficiency for NAU codons. Thus, queuosine has the capacity to suppress the initial codon usage bias, exhibiting a 20% decrease in NAU decoding in the absence of Q34 and a 34% increase in over-modified tRNAs [63]. However, the opposite has been demonstrated in *V. cholerae,* particularly for UAU codons of tyrosine that are more efficiently decoded compared to UAC codons in absence of queuosine [64]. Among the other examples, the absence of the MiaA enzyme, responsible of i^6^A37 modification, alters the decoding of UXX and UUA codons in *Streptomyces albus* (*S. albus*) [65] and its presence is required for the correct decoding of Leu-UUX codons in *E. coli* [66]. Still in *E. coli*, the s^2^U34 modification enhances the binding affinity of the tRNA-Gln for CAA and CAG codons [62], and the mnm^5^s^2^U34 modification on tRNA-Lys(UUU) stabilizes non-canonical base pairing [67]. In Mycobacterium strains, the increase of cmo^5^U-modified tRNA-Thr(UGC) induces a preferential translation of ACG over ACA and ACU codons [68]. Moreover, it has been demonstrated that a *gidA* (now named *mnmG*) mutant of *P. aeruginosa*, deficient for cmnm^5^U34 modification, shows a decrease of translation efficiency for Arg-AGA and Leu-UUA codons that are rare codons [69].

One way for tRNA modifications to enhance decoding efficiency, without favoring specific codons, is by increasing codon degeneracy. For instance, the TadA enzyme catalyzes the deamination of adenosine at position 34 of tRNA-Arg(ACG) into inosine, and this modified nucleotide is then read as a guanosine in sequencing reactions. The presence of inosine facilitates the recognition of CGA, CGU, and CGC codons, and induces a more efficient translation of arginine codons [70]. Furthermore, this extension of codon degeneracy allows the bacteria to reduce the requirement of tRNA-genes [71].

It is worth noting that while tRNA modifications predominantly have a positive influence on translation by affecting structure, fidelity, and efficiency, certain modifications have the potential to exert undesirable effects. In *E. coli*, mnm^5^s^2^U and ψ modifications respectively induce misreading of aspartate and histidine codons, creating mistranslation errors [72,73].

### How modifications provide a quality control for tRNAs?

Given that RNA modifications are essential for the formation and stabilization of tRNA molecules, the status and extent of these modifications can serve as a quality control mechanism for tRNAs. In eukaryotes, the recognition and elimination of hypo- or hyper-modified tRNAs is facilitated by specific RNA degradation systems, including the nuclear surveillance pathway and the rapid tRNA decay pathway. For example, the absence of certain tRNA modifications act as a signal molecule for these two systems in eukaryotes [30]. In *Vibrio cholerae*, the absence of s^4^U modification in the elbow of tRNAs results in the decay of hypo-modified tRNAs by the RNA degradosome system [74]. Another way to ensure tRNAs quality control has been described in *E. coli* where the presence of m^5^U54 contributes to tRNA maturation as its absence is associated with an altered modifications pattern and impaired ribosome translocation [75].

## Impact of tRNA modifications on bacterial virulence

Bacteria are well known for adapting their lifestyle to the environment. Specifically, when considering pathogenic bacteria, their ability to respond to stress and switch to a state of virulence is key for manipulating, killing, and/or surviving within theirs hosts [1,2]. These aspects involve numerous proteins and effectors that must be finely regulated to ensure that each virulence factors are expressed at the right place and time during infection. As described above, one way of regulating gene expression is at the translation level. Additionally, the modulation of translation fidelity is imperative under stress exposure and during host interaction as, under optimal conditions, amino acid misincorporation is around 0.01% and the readthrough frequency around 0.5%; whereas under stressful conditions, the misincorporation rate increases to 1% and readthrough errors to 10% [76].

Considering the significant and varied impacts of tRNA modifications on the translation process and the translation of specific codons, their influence on the production of stress responses and virulence factors in pathogenic bacteria is of particular interest.

### Are tRNA modifications essential?

**Fig 2:**
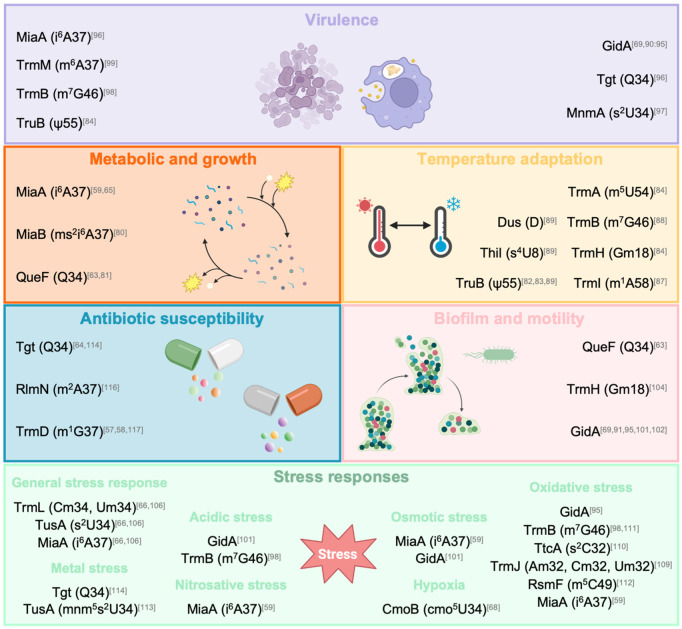
tRNA modifications drive bacterial virulence and stress adaptation. The effects of the tRNA modifications on bacterial physiology mentioned in this review are listed under the six categories described. The modifying enzymes are listed with their modifications in parentheses, and the related bibliographic references are indicated with superscripts. The illustrations in this figure were created using BioRender: Moreau, K. (2025) https://BioRender.com/3ohwa91.

Most enzymes responsible for tRNA modifications are not essential under optimal growth laboratory conditions and a significant number of phenotypes linked to tRNA modifications have been discovered under stress conditions, suggesting a strong implication in cellular fitness despite their non-essential nature [49] (Fig 2 and S1 Table).

Nevertheless, there are tRNA-modifying enzymes that are essential in the model bacteria *E. coli* and *B. subtilis*. They mostly modify the ASL: TilS (k^2^C34), TsaBCDE (t^6^A37), and TrmD (m^1^G37) [39]. Enzymes can also be essential only for some species such as TadA (I34) that is essential for *E. coli* and *B. subtilis* but not for *S. aureus* [21,77].

Moreover, certain tRNA modifications have synergistic effects as double knockout mutants showed an exacerbation of the phenotype observed in single knockout mutants. For example, in *E. coli*, five pairs of modifications were identified as essential, as their double knockout mutants exhibited no growth. Among these five pairs, which represents 1% of the tested combinations, four include ASL-modifying enzymes. Notably, TruA is the most frequent, catalyzing the ψ39 and ψ40 modifications that serve as quality control of tRNAs. More generally, it is important to note that the lethality of the tested pair depends on the growth conditions [78]. The requirement for tRNA modifications depends on conditions such as culture medium, temperature, and substrate. For instance, levels of queuosine modification in *Streptococcus mutans* (*S. mutans*) are influenced by medium composition with the micronutrients content [79].

### How do tRNA modifications alter growth and metabolism?

Metabolism is a key determinant of virulence as it allows bacteria to adapt and persist within its host. In ExPEC strains, the MiaA (i^6^A37) enzyme is finely regulated as it modulates frameshift level to acquire metabolic flexibility *in vivo*. This regulatory process creates a fitness advantage during gut colonization, urinary tract infections, and bloodstream infections [59]. MiaA is also involved in morphogenesis of certain bacteria as its inactivation leads to delayed hyphal development and spore formation in Streptomyces [65]. Moreover, in *Streptomyces ghanaensis* (*S. ghanaensis*), ms^2^i^6^A37 modification by MiaB, which occurs following MiaA modification, modulates morphology development by increasing translation efficiency of rare codon Leu-UAA in life cycle genes [80]. Queuosine (Q34) modification is also implicated in metabolism, as overexpression of *queF* confers a fitness advantage for *P. putida* over *E. coli* [63] and its absence reduces the viability of *E. coli* during the stationary phase [81].

### How do tRNA modifications allow bacteria to adapt to temperature?

During growth or infection, bacteria may encounter varying temperatures, thus it is crucial for them to respond effectively to these temperature shifts. Most of the tRNA modifications involved in this aspect are in the arms of tRNAs and have an impact on structure maintenance. Ψ55 modification made by the TruB enzyme is critical for the survival of *E. coli* at temperatures over 37 °C. Deficiency in this modification alters significantly the proteome, particularly affecting outer-|membrane proteins [82]. To better understand the role of tRNA modifications in temperature adaptation, various studies have been conducted on nonpathogenic thermophilic species. In *Thermus thermophilus* (*T. thermophilus*), TruB is essential for adaptation to low temperatures, as the Δ*truB* mutant presents an impaired growth at 55 °C. Moreover, TruB regulates the amount of other tRNA modifications at 55 °C such as Gm18, m^5^s^2^U54, and m^1^A58, therefore impacting tRNA structure and melting temperature [83]. Certain of these modifications contribute also to the survival of *E. coli* at high temperatures including Gm18 by the TrmH enzyme and m^5^U54 by the TrmA enzyme [84].

Thermophilic bacteria require appropriate cellular systems for growth in hot environments. RNA modifications play a role in this resistance and, interestingly, some of these modifications are even specific to thermophilic species. For instance, the m^5^s^2^U54 modification is exclusively found on the tRNAs of thermophiles and is essential for protein production at high temperature by increasing the melting temperature of tRNAs [85]. Its content is finely regulated to ensure a balance to adapt growth to different temperature gradients. At low temperatures, there is a higher proportion of m^5^U54 compared to m^5^s^2^U54, while at high temperatures, the opposite is found [86]. Other modifications, not specific to thermophilic bacteria, have been also described as essential for *T. thermophilus* growth at high temperatures such as m^1^A58 by the TrmI enzyme at temperatures above 80 °C [87] and m^7^G46 by the TrmB enzyme at temperatures above 70 °C [88].

A comparison of RNA modifications presents in psychrophiles, mesophiles, and thermophiles belonging to the Bacillales at minimal, optimal, and maximal temperatures reveals distinct profiles. In thermophiles, the amount of s^4^U8 and Ψ55 modifications increase under optimal growth temperature to confer more rigidity to the tRNA structure. In contrast, at minimal growth temperature for mesophiles and psychrophiles, only an increase of dihydrouridine modifications is observed which allows a more flexible tRNA structure to survive in cold environment [89].

### How do tRNA modifications modulate the virulence levels of bacteria?

Bacterial virulence is illustrated by numerous aspects, such as infection capacity, cytotoxicity, and survival in animal models. GidA (MnmG), that catalyzes multiple tRNA modifications, has been identified as a key enzyme involved in the regulation of overall virulence in various pathogenic bacteria. For instance, in *Streptococcus pyogenes* (*S. pyogenes*), its inactivation reduces protease expression, affecting the global proteome and decreases the translation of virulence factors, thus reducing virulence in the murine ulcer model [90]. In *Salmonella enterica* (*S. enterica*), a similar impact was observed as the lack of the GidA (MnmG) enzyme leads to pleiotropic phenotypes ultimately illustrated by a decrease of cytotoxicity and lower intracellular survival in the murine model [91]. GidA (MnmG) also promotes virulence via directly enhancing translation of bacterial toxins such as cytotoxic necrotizing factor 1 (CNF1) in *E. coli* [92] and Act in the food pathogen *Aeromonas hydrophola* (*A. hydrophola*) [93]. Several studies have also demonstrated the implication of the GidA (MnmG) enzyme in the virulence of *P. aeruginosa*, *in vitro* and *in vivo*. Firstly, Gupta *and colleagues* observed in 2009 that GidA (MnmG) impacts quorum-sensing gene expression by regulating the translation efficiency of RhlR, one of the two major quorum-sensing systems [94]. Then, Srimahaeak *and colleagues* reported in 2023 that GidA (MnmG) is required for the full bacterial virulence in macrophages *in vitro* and in **Caenorhabditis elegans* in vivo* [95]. Lastly, Krueger *and colleagues* reported in 2024, that the absence of GidA (MnmG) results in an avirulent *P. aeruginosa* strain in the *Galleria mellonella* model and exhibiting a fitness disadvantage during mouse infection due to a reduced cytotoxicity against macrophages [69].

While the role of GidA (MnmG) in bacterial virulence is evident, it is not the only one. Several other enzymes also play crucial roles in modulating bacterial infection. For instance, in *Shigella flexneri* (*S. flexneri*), MiaA and Tgt enzymes favor translation of the major regulator VirF to increase pathogenicity [96] and the absence of TruB enzyme reduces the expression of several virulence genes [84]. Furthermore, the presence of s^2^U34 modification, made by the MnmA enzyme, in *Mycobacterium tuberculosis* (*M. tuberculosis*) enhances intracellular growth in macrophages. Interestingly, this benefit was not observed under optimal growth conditions, indicating that this modification is only required for growth in infectious contexts [97]. Another example is the TrmB (m^7^G46) enzyme that promotes *Acinetobacter baumannii* (*A. baumannii*) adaptation, as its absence results in decreased virulence in mice and reduced replication within macrophages [98].

Interestingly, bacteria can also use regulation of tRNA modifications to prevent phage infection. It has been described for the first time that the lack of m^6^A modification in *E. coli* reduces the infection efficiency of T5 phage [99].

### How do tRNA modifications alter the formation of biofilm and bacterial motility?

Biofilm is a strategy employed by bacteria to survive in hostile environments, by adhering to a surface and aggregating within an extracellular matrix. This process enables the bacterial population to evade the immune system and antimicrobial agents, making it a crucial aspect of bacterial virulence [100]. As a key component of bacterial pathogenicity, biofilm is also impacted by tRNA modifications. For instance, biofilm formation is stimulated by Q34 modification that increases bacterial aggregation in *E. coli* and teichoic acid production in *B. subtilis* [63]. The inactivation of GidA (MnmG) or MnmE enzymes in *S. mutans* also reduces glucose-dependent biofilm biomass by 50% due to impaired initial surface attachment [101]. Similarly, GidA (MnmG) influences biofilm formation in *P. aeruginosa*, where the absence of cmnm^5^U34 modification alters biofilm structure, leading to more dead bacteria at the base and an overall reduction in the amount of biofilm [69,95].

Motility is another advantage during infection as it allows bacteria to disseminate within the host. As for biofilm formation, the GidA (MnmG) enzyme stimulates bacterial motility by improving translation efficiency of flagella proteins of *P. aeruginosa* [69,95]. Likewise, GidA (MnmG) inactivation decreases *S. enterica* motility by a factor of two [91,102]. Two other tRNA-modifying enzymes, TadA and TrmH, are implicated in bacterial motility regulation. TadA usually modifies A34 to I34; however, in the rice pathogen *Xanthomonas oryzae*, *tadA* induction after H_2_O_2_ stress exposure, increases S128C mutation in FliC, which modifies flagellar structure and increases motility [103]. In contrast, absence of Gm18 modification made by TrmH in *E. coli* enhances expression of motility genes, associated with an increase of swarming [104].

### How do tRNA modifications impact stress responses?

Bacteria are exposed to different stress during infection such as oxidative and osmotic stresses, variation of pH, metal starvation or excess, and antibiotics [105]. They have developed many strategies to adapt their gene expression in response to these exposures, with tRNA modifications being a notable example. In *E. coli*, MiaA (i^6^A37), TrmL (Cm34 and Um34), and TusA (s^2^U34) enzymes allow the translation of the major regulator of stress response, the sigma factor RpoS. Indeed, RpoS possess a bias toward the Leu-UUX codon, necessitating Cm/Um34 and s^2^U modifications for efficient and accurate translation. As Cm34/Um34 only occur when i^6^A37 is present on tRNAs, all three modifications are required for the translation of the sigma factor [66,106]. In a similar manner, i^6^A37 supports the correct translation of another major regulator, Hfq chaperon protein [106]. MiaA in ExPEC strains is also involved in various stress responses as the presence of the enzyme stimulates growth under nitrosative, oxidative, hyper- and hypo-osmotic conditions [59], possibly due to its influence on the translation of RpoS and Hfq.

Reactive oxygen species (ROS) are the products of oxidative stress and alters biomolecules to impact metabolism, causing significant cellular damage [107]. Bacteria use diverse systems to counteract ROS, and RNA is recognized as a central signaling molecule [108]. Several modifying enzymes, such as TrmJ (Am32, Cm32, and Um32) and TtcA (s^2^C32), have been described to favor *P. aeruginosa* resistance to H_2_O_2_ exposure by up-regulating expression and activity of catalase. Furthermore, *ttcA* is upregulated under oxidative stress [109,110]. TrmB is also essential for bacterial survival under oxidative stress as its modification, m^7^G46, is raised following H_2_O_2_ exposure, leading to the increase of translation efficiency of specific codons present in the catalases KatA and KatB [111]. A comparable effect on the translation of these catalases has been observed for GidA (MnmG) and mnm^5^U34 modification [95]. Another example can be found in *A. baumannii*, in which a decrease in m^7^G46 modification impairs growth under oxidative stress by affecting translation. This modification enhances the translation of ion transport and metabolic proteins, while its absence promotes the translation of transcription and replication proteins to compensate for altered protein production [98]. More generally, all-but-three RNA modifications decrease after oxidative stress exposure in *E. coli*. One of these three, m^5^C49, rises under stress, accompanied by an upregulation of its modifying enzyme RsmF, enhancing bacterial resistance to oxidative stress [112].

Regarding metal stress, mnm^5^s^2^U34 modification catalyzed by the TusA enzyme in *E. coli* promotes translation of the Fur regulator that represses expression of siderophore genes and activates those involved in iron storage [113]. Additionally, Q34 modification is responsible for pleiotropic effects regarding metal stress as its absence enhances resistance to nickel and cobalt as well as sensitivity to cadmium [114]. Under osmotic and acidic stress inactivation of the GidA (MnmG) enzyme reduces growth of *S. mutans* [101]. The same outcome under acidic stress has been observed in the absence of TrmB as the lack of the enzyme leads do a reduction of *A. baumannii* proliferation at pH5, associated with decrease in replication in macrophages [98]. Another type of stress encountered during infection is hypoxia. For *Mycobacterium bovis* (*M. bovis*), the major regulator of hypoxia, DosR, is enriched in codons ACG that need cmo^5^U modification on tRNA-Thr(UGU) to be efficiently translated. Intriguingly, the amount of cmo^5^U and tRNA-Thr(UGU) increases under hypoxia [68].

### How do tRNA modifications influence antibiotic susceptibility?

Antibiotic resistance is a major problem in healthcare. Pathogens are rapidly developing new resistance mechanisms, which outpace the development of new drugs, making infections more difficult to treat. In *V. cholerae* and, to a lesser extent in *E. coli*, tRNA and rRNA modifications impact antibiotic resistance and growth under sub-inhibitory concentrations of various antibiotic classes. However, the effects observed vary according to the antibiotic, the enzyme, and the species [115]. Queuosine is one of the modifications involved in aminoglycoside tolerance in *V. cholerae*, as *tgt* inactivation results in a growth defect under sub-minimal inhibitory concentrations of tobramycin, and *tgt* is overexpressed under similar stress conditions [64]. RsxA is a stress response inhibitor that reduces bacterial survival and is enriched in Tyr-TAT codons (83% vs. 53% on average) and, conversely, housekeeping genes are more prone to a TAC bias, which require Q34 for an efficient translation. Therefore, Tgt and Q34 promote bacterial survival under tobramycin stress by reducing the translation of RsxA and increasing that of housekeeping proteins [64]. In addition, Q34 modification has been shown to favor resistance to aminoglycosides in *E. coli* [114].

In *Enterococcus faecalis* (*E. faecalis*), the RlmN enzyme, which modifies both tRNA and 23S rRNA, plays a role in antibiotic resistance as a ROS sensor and immediately regulates the translation of stress response proteins. Overexpression of *rlmN* increases sensitivity to ampicillin and ciprofloxacin, while its absence leads to resistance to chloramphenicol [116].

Another modification involved in response to antibiotic exposure is m^1^G37 made by TrmD, especially in *E. coli* and *S. enterica* [117]. This methylation modulates translation by promoting the decoding of CCC and CCU proline codons. In the absence of modification, ribosomes stall on mRNA, inducing a frameshift or premature termination, which can result in cellular death. Membrane-associated proteins in Gram-negative bacteria are enriched in these proline codons due to the structure requirement of the transmembrane [57,58]. A decrease in m^1^G37 has been shown to impair the outer membrane structure and envelope integrity in *E. coli* and Salmonella, thus making the bacteria more susceptible to various classes of antibiotics, including beta-lactams, aminoglycosides, and quinolones [117].

## Conclusion and perspectives

Organisms from the three domains of life harbor tRNA modifications, and some of these are more conserved than others. The core body of tRNAs, i.e., the elbows and arms, exhibit a high degree of conservation in modification functions, attributable to their impact in tRNAs structural rigidity and stability. In contrast, ASL modifications have a lower degree of conservation among organisms, suggesting that these modifications may play a role in adaptation [49]. Together, all tRNA modifications modulate translation process by influencing tRNA structure, reading frame maintenance, stop codon readthrough, as well as the accuracy and efficiency of codon decoding. However, despite many studies reviewed here reporting the impact of tRNA modifications on virulence and stress responses, the majority do not investigate the molecular mechanism underlying these phenotypes. Most studies rely on mutants lacking a single tRNA-modifying enzyme and demonstrate that its absence results in specific phenotypes, without establishing the precise link between the modification, translation alteration, and the phenotype. This gap remains a key challenge in the field and should be addressed by future studies to clarify how tRNA modifications mediate their effects.

Despite the identification of the first RNA modification in yeast 70 years ago [9], it is only recently, with advancements in detection methods, that the first mapping of tRNA modifications has been achieved in model bacteria [5,39]. The improvement of sequencing methods, bioinformatics tools, and analytical techniques such as mass spectrometry coupled with liquid chromatography or single-cell RNA sequencing, now enable the precise study of dynamic regulation, as is the case for RNA modifications [86]. As mentioned in the 2024 NASEM report, the comprehensive mapping of RNA modifications in bacteria is still in its early stages [118].

While the primary function of tRNA-modifying enzymes is catalytic, these enzymes can also play additional roles within the cell. For example, some modifying enzymes act as chaperones [30]. Therefore, deletion of such enzymes may not only affect tRNA modification but may also impact other cellular functions. This highlights the potential link between tRNA modifications and cellular processes, suggesting that perturbation of these enzymes could have far-reaching effects beyond their primary role.

As demonstrated in the present review, tRNA modifications are essential for environmental adaptation and virulence without requiring protein synthesis [85]. The regulatory mechanism involving tRNA modifications can occur in two distinct ways: either directly, by modulating the expression of virulence factors, or indirectly, by targeting a regulator upstream of the virulence factor. Additionally, some modifications have pleiotropic effects by affecting a global regulator or causing protein aggregation through misfolding [10]. Considering the significant and varied impacts of tRNA modifications on the translation process, and given that modulating translation fidelity is crucial for bacterial stress responses and host interactions [76], these modifications need to be finely tuned for precise regulation; an aspect that has yet to be fully explored. One strategy to achieve this is the turnover of modified tRNAs through degradation and *de novo* synthesis. However, another quicker solution relies on the notion of reversibility of tRNA modifications. Therefore, as modifying enzymes exist (known as writers), de-modifying enzymes also exist (known as erasers) to regulate modification levels. These enzymes are responsible for specific de-modification on tRNA by converting the modified nucleotide into its initial form. Erasers are primarily described in eukaryotes [119], but two examples have also been described in bacteria. Firstly, the RudS de-modifying enzyme catalyzes the conversion of s^4^U8 to uridine. Several RudS orthologs from *P. putida*, *S. enterica* and different strains of *E. coli* have been tested, and all reduced the level of s^4^U8 modification in *E. coli* [120]. Secondly, the two erasers RMD1 and RMD2, have been identified in *Streptomyces venezulae* in which they regulate the amount of m^1^A58 modification in function of environmental conditions [121]. Nevertheless, with only these two examples reported in the literature to date, the concept of erasers and the role of de-modifying enzymes in bacterial adaptation remain largely unexplored. It is essential to first identify new eraser candidates and then to investigate their impact on bacterial virulence and stress responses.

As tRNA modifications play a role in the development of antibiotic resistance [57,58,64,115,117], it is important to exploit this aspect to develop new drugs targeting RNA-modifying enzymes. For instance, a peptide was designed to mimic the α-helix structure required for YeaZ dimer formation, a protein involved in t^6^A37 modification synthesis. The initial 14-amino acid peptide (PMP1) was optimized into PMP3 by adding a cell-penetrating peptide. PMP3 was able to enter *P. aeruginosa* cells and slightly reduce growth. However, in *E. coli*, the effect was much weaker, likely due to structural differences in YeaZ [122]. This result highlights the species specificity of protein-protein interaction (PPI) modulators and suggests that broad-spectrum applications would require targeting conserved motifs or developing species-specific compounds [122]. Another illustration of this application is the development of TrmD (m^1^G37) inhibitors as this modifying enzyme is conserved and essential in various bacterial pathogens while its human homologue presents a different structure. Thanks to a high-throughput screening method, a library of 116,350 small molecules was tested for their inhibitory action against the methyltransferase activity of TrmD. Five molecules were identified as being active against Gram-positive bacteria and causing a growth inhibition of *M. bovis*, *Mycobacterium smegmatis*, *S. aureus*, *Streptococcus pneumoniae* or *E. faecalis*, with one demonstrating a more global antibacterial activity against all of these species [123]. In a subsequent study, the same authors aimed to elucidate the mechanism of action of these compounds and to expand their activity towards Gram-negative bacteria before antibiotic development. They synthetized different thienopyrimidinone derivatives, guided by the findings of their first study and the TrmD crystal structure of *P. aeruginosa* and *M. tuberculosis* [124]. Some of these derivatives interfered with TrmD enzymatic activity by preventing the binding of the cofactor or the tRNA substrate. Three of the identified compounds exhibited antibacterial activity not only against Gram-positive but also against Gram-negative bacteria by inhibiting growth of *A. baumannii* and *Salmonella enteritidis* [124]. In addition, small molecules inhibiting Tgt (Q34) have also been produced, but their antibacterial activity remains to be tested. For example, *lin*-benzoguanine derivatives were synthesized and their inhibitory activity against Tgt was evaluated. These molecules act as nucleobase analogs, competing with tRNA for binding to Tgt by occupying the PreQ1 pocket [125]. Nevertheless, extensive testing is still required for these three examples to be further developed as antibiotics. Specifically, investigations of *in vivo* stability, human cell toxicity, and the potential emergence of resistance must be conducted. It is also essential to ensure that these molecules do not target human RNA-modifying enzymes.

Another clinical application of tRNA modifications could be to detect bacterial infection by quantifying the amount of tRNA modifications specific to bacteria such as m^2^A modification. For example, m^2^A levels increase after antibiotic treatment or immune cells exposure, indicating that this modification is released after bacterial lysis. Furthermore, m^2^A is detected in mouse urine after m^2^A injection or *E. coli* infection, and thus m^2^A could be used as a signaling molecule to detect bacterial infection in urine samples [31]. Although the m^2^A tRNA modification is not specific to pathogenic bacteria, a rise in amount may be an alert for further investigation.

In conclusion, although the study of tRNA modifications in bacteria is still in its infancy, their central role in adaptation, virulence, and antibiotic resistance highlights an exciting field, where future discoveries could lead to fine-tuned regulatory mechanisms and therapeutic applications.

## Supporting information

S1 Table Summary of the impacts of tRNA modifications on bacterial virulence and stress adaptation. The table summarizes the data discussed throughout the study and illustrated in Fig 2. The tRNA modifications and the associated enzymes, the species studied, and the impacts observed in a mutant lacking the enzyme are listed.
(DOCX)
