# Supplementary material for: tRNA modifications as regulators of bacterial virulence and stress responses
Source: PLoS Pathog. 2025 Oct 23;21(10):e1013600. doi: 10.1371/journal.ppat.1013600 (PMC12548931; doi:10.1371/journal.ppat.1013600)
Supplement: S1 Table — The table summarizes the data discussed throughout the study and illustrated in Fig 2. The tRNA modifications and the associated enzymes, the species studied, and the impacts observed in a mutant lacking the enzyme are listed. (DOCX) [file ppat.1013600.s001.docx]

**Table S1: Summary of the impacts of tRNA modifications on bacterial virulence and stress adaptation.**

The table summarizes the data discussed throughout the study and illustrated in Fig 2. The tRNA modifications and the associated enzymes, the species studied, and the impacts observed in a mutant lacking the enzyme are listed.

|  | **Enzyme** | **Modification** | **Bacterial species** | **Phenotypes in mutant** | **References** |
| --- | --- | --- | --- | --- | --- |
| **Growth and metabolism** | MiaA | i^6^A37 | ExPEC | Loss of metabolic flexibility and fitness advantage during gut colonization, urinary tract or bloodstream infections. | 59 |
|  |  |  | *S. albus* | Delayed hyphal development and spore formation. | 65 |
|  | MiaB | ms^2^i^6^A37 | *S. ghanaensis* | Impaired spore formation. | 80 |
|  | QueF | Q34 | *P. putida* | Decreased fitness advantage against *E. coli*. | 63 |
|  |  |  | *E. coli* | Reduced viability during stationary phase. | 81 |
| **Temperature adaptation** | TruB | ψ55 | *E. coli* | Less survival over 37°C. | 82 |
|  |  |  | *T. thermophilus* | Less adapted to low temperatures: impaired growth at 55°C. | 83 |
|  |  |  | *G. stearothermophilus* | Increase in the amount of the modification at the optimal growth temperature. | 89 |
|  | TrmA | m^5^U54 | *E. coli* | Less survival at high temperatures. | 84 |
|  | TrmB | m^7^G46 | *T. thermophilus* | Less survival at high temperatures (70°C). | 88 |
|  | TrmH | Gm18 | *E. coli* | Less survival at high temperatures. | 84 |
|  | TrmI | m^1^A58 | *T. thermophilus* | Less survival at extreme temperature (80°C). | 87 |
|  | Dus | D16 | *B. subtilis* | Increase in the amount of the modification at low temperature. | 89 |
|  | Dus | D47 | *E. sibiricum* | Increase in the amount of the modification at low temperature. | 89 |
|  | ThiI | s^4^U8 | *G. stearothermophilus* | Increase in the amount of the modification at the optimal growth temperature. | 89 |
| **Virulence** | MiaA | i^6^A37 | *S. flexneri* | Reduced global virulence and lower expression of virulence regulator. | 96 |
|  | Tgt | Q34 |  |  |  |
|  | TrmM | m^6^A37 | *E. coli* | Reduced infection efficiency of T5 phage. | 99 |
|  | TrmB | m^7^G46 | *A. baumannii* | Reduced replication in macrophages and decreased virulence in murine model. | 98 |
|  | TruB | ψ55 | *S. flexneri* | Reduced expression of virulence factors. | 84 |
|  | GidA | - | *P. aeruginosa* | Reduced cytotoxicity against murine macrophages resulting in avirulent strain. | 69 |
|  |  |  | *S. pyogenes* | Reduced virulence in murine ulcer model. | 90 |
|  |  |  | *S. enterica* | Reduced virulence in the murine model, invasion of gut epithelial cells, intracellular survival and cytotoxicity in macrophages. | 91 |
|  |  |  | *E. coli* | Lower production of bacterial toxin. | 92 |
|  |  |  | *A. hydrophola* | Lower production of bacterial toxin. | 93 |
|  |  |  | *P. aeruginosa* | Altered expression of quorum sensing system. | 94 |
|  |  |  | *P. aeruginosa* | Reduced bacterial virulence *in vitro* and *in vivo*. | 95 |
|  | MnmA | s^2^U34 | *M. tuberculosis* | Decreased intracellular growth in macrophages. | 97 |
| **Biofilm and motility** | QueF | Q34 | *E. coli* | Lower biofilm formation via bacterial aggregation alteration. | 63 |
|  |  |  | *B. subtilis* | Lower biofilm formation via teichoic acid production alteration. |  |
|  | TrmH | Gm18 | *E. coli* | Increased swarming capacity. | 104 |
|  | GidA | - | *P. aeruginosa* | Altered biofilm structure and amount. | 69, 95 |
|  |  |  | *S. enterica* | Decreased motility. | 91, 102 |
|  |  |  | *P. aeruginosa* | Decreased motility via lower production of flagella protein. | 69, 95 |
|  |  |  | *S. mutans* | Reduced biofilm formation due to impaired initial surface attachment. | 101 |
| **Stress responses** | TrmL | Cm34, Um34 | *E. coli* | Altered translation of stress response regulator. | 66,106 |
|  | TusA | s^2^U34 |  |  |  |
|  | Tgt | Q34 | *E. coli* | Increased resistance to nickel and cobalt as well as sensitivity to cadmium. | 114 |
|  | GidA | - | *P. aeruginosa* | Lower survival under oxidative stress due to reduced catalase activity. | 95 |
|  |  |  | *S. mutans* | Reduced growth under osmotic and acidic stress. | 101 |
|  | TusA | mnm^5^S^2^U34 | *E. coli* | Altered iron homeostasis and iron sulfur cluster assembly. | 113 |
|  | CmoB | cmo^5^U34 | *M. bovis* | Lower survival under hypoxia due to altered regulator expression. | 68 |
|  | TrmJ | Am32, Cm32, Um32 | *P. aeruginosa* | Lower resistance to H_2_O_2_ exposure due to reduced catalase activity. | 109 |
|  | TtcA | s^2^C32 | *P. aeruginosa* | Lower resistance to H_2_O_2_ exposure due to reduced catalase activity. | 110 |
|  | MiaA | i^6^A37 | *ExPEC* | Lower growth under nitrosative, oxidative, hyper- and hypo-osmotic conditions. | 59 |
|  |  |  | *E. coli* | Incorrect translation of stress response regulator. | 66, 106 |
|  | TrmB | m^7^G46 | *A. baumannii* | Lower resistance to oxidative and acidic stress. | 98 |
|  |  |  | *P. aeruginosa* | Lower survival under oxidative stress due to reduced catalase activity. | 111 |
|  | RsmF | m^5^C49 | *E. coli* | Lower resistance oxidative stress resistance. | 112 |
| **Antibiotic susceptibility** | Tgt | Q34 | *V. cholerae* | Growth defect under tobramycin stress. | 64 |
|  |  |  | *E. coli* | Lower resistance to aminoglycosides. | 114 |
|  | RlmN | m^2^A37 | *E. faecalis* | Increased sensitivity to ampicillin and ciprofloxacin as well as resistance to chloramphenicol. | 116 |
|  | TrmD | m^1^G37 | *E. coli*, *S. enterica* | Increased sensitivity to different antibiotics via membrane structure alteration. | 57, 58, 117 |
